# Compared with High-intensity Interval Exercise, Moderate Intensity Constant Load Exercise is more effective in curbing the Growth and Metastasis of Lung Cancer

**DOI:** 10.7150/jca.66245

**Published:** 2022-02-28

**Authors:** Zhe Ge, Shan Wu, Zhengtang Qi, Shuzhe Ding

**Affiliations:** 1 School of Sport, Shenzhen University, Shenzhen 518060, China.; 2 Key Laboratory of Adolescent Health Assessment and Exercise Intervention of Ministry of Education, East China Normal University, Shanghai 200241, China.

**Keywords:** exercise, angiogenesis, lymphangiogenesis, EMT, MMP2/9, Lung cancer

## Abstract

The morbidity and mortality of lung cancer are among the forefront of various cancers, and it is one of the major cancers that seriously threaten human life and health. It is well known that the abundant angiogenesis and lymphangiogenesis in tumor tissues play an important role in tumor growth and metastasis. In addition, The epithelial-mesenchymal transition (EMT), which facilitates the tumor cell metastasis and invasion, is triggered by many stimuli, such as matrix metalloproteinases 2 (MMP2), MMP9, and transforming growth factor-β1 (TGF-β1). At present, various studies have confirmed that both moderate intensity constant load exercise (MICE) and high-intensity interval exercise (HIIE) have a positive therapeutic effect on the treatment of lung cancer, delaying the progression of lung cancer. However, little is currently known regarding whether its specific treatment mechanism is related to blood vessels, lymphatic vessels, and EMT. Indeed, we found an increase in angiogenesis and lymphangiogenesis in lung cancer tissues. However, compared to high-intensity interval exercise, moderate intensity constant load exercise can significantly reduce tumor growth in the lung independent of blood vessels and lymphatic vessels. It is worth noting that moderate intensity constant load exercise can also reduce the level of MMP9 in lung cancer tissues, which may control tumor metastasis to a certain extent. In addition, high-intensity interval exercise reduces the expression of MMP2, but it tends to enhance EMT and activate TGF-β1. Taken together, our findings suggest that, whether it is tumor growth or metastasis, moderate intensity constant load exercise has a better therapeutic effect on lung cancer than high-intensity interval exercise.

## Introduction

Malignant tumor is one of the principal diseases threatening human health. Due to the increasing aging of the global population, smoking, overweight, lack of physical activity, and other factors, the burden of cancer incidence and mortality is rapidly growing worldwide 1. Lung cancer is one of the most common malignant tumors in the world, with high morbidity and mortality. According to the global cancer statistics in 2020, the incidence of lung cancer is the second highest among all major cancers, after breast cancer, and the death rate of lung cancer is the highest among all major cancers 2. It has been demonstrated that exercise can reduce the incidence of esophageal cancer by 42%, the incidence of liver cancer by 27%, and the incidence of lung cancer by 26% 3. Moreover, the symptoms of lung cancer patients have improved after the aerobic exercise intervention recommended by the therapist 4. Besides, it has been proven that high-intensity interval exercise also delays the progression of lung cancer 5. Notably, angiogenesis and lymphangiogenesis in tumor tissues are conducive to the growth and metastasis of tumor cells 6. Epithelial-mesenchymal transition (EMT) is the transformation process of epithelial cells to mesenchymal cells, which plays an important role in development, wound healing and various diseases. However, EMT in cancer cells is thought to enhance their invasive properties 7. Mechanistically, the EMT that triggers cellular mobility and subsequent dissemination of tumor cells is activated by matrix metalloproteinases 2 (MMP2), MMP9, transforming growth factor-β1 (TGF-β1), and collagen type I 8-11, which are also beneficial to tumor angiogenesis 12. MMP2 and MMP9 that play a role in tumor invasion and metastasis are proteolytic enzymes involved with extracellular matrix (ECM) degradation 13. Collagen type I, the major extracellular matrix component of skin and bone, are associated with epithelial-to-mesenchymal transition in cancer cells 14. Furthermore, it is well known that TGF-β1 signaling events control a variety of processes and numerous responses, such as cell proliferation, differentiation, apoptosis, and migration. In healthy cells and pre-malignant cells, TGF-β1 primarily functions as a tumor suppressor, while in the later stage of cancer, TGF-β1 signaling promotes tumor invasion and metastasis 15. Additionally, TGF-β1 derived from tumor cells can promote tumor growth by triggering angiogenesis, EMT, and MMP system degradation of ECM 16. However, whether moderate intensity constant load exercise (MICE) or high-intensity interval exercise (HIIE) can inhibit tumor growth and metastasis through these pathways remains unclear. Thus, the current study mainly explores the mechanism by which exercise inhibits tumor growth and metastasis from these perspectives.

## Material and methods

### Animals and groups

6-week-old specific-pathogen-free (SPF) grade BALB/c female mice were purchased from the Experimental Animal Center of East China Normal University. All mice were housed in a temperature-controlled (21-22 °C) SPF laboratory animal room under 12-hr light-dark cycles with access to food and water *ad libitum*. The mice were randomly divided into four groups: saline group (Saline, n=10), urethane control group (CON, n=25), moderate intensity constant load exercise group (MICE, n=15), high-intensity interval exercise Group (HIIE, n=15). Except for the saline group (Saline), all mice received 600 mg/kg intraperitoneal injection of urethane (94300, Sigma-Aldrich) once a week for 10 consecutive weeks. The saline group was injected with equal volume of normal saline.

### Exercise Model

All mice continued to be fed for 15 weeks after intraperitoneal injection of urethane for 10 consecutive weeks. Then 10 mice were randomly taken out from the CON group and sacrificed, and lung tissues were taken to observe whether the mouse lung cancer model was successful. After confirming that all mice were successfully modeled, the mice in the MICE group and the HIIE group performed 12 weeks of exercise. The MICE group performed aerobic endurance exercise with an exercise intensity of 15m/min (80% VO2max) for 45 minutes, 5 times a week 17. The HIIE group performed high-intensity interval swimming exercises with a plastic water tank of 40 x 30 x 80 cm and a water temperature of 30±2 °C. A lead mass of 10% or 12% of body weight was suspended from the tail of the mouse, forced to swim for 20 seconds, and then passively recovered for 10 seconds, repeated 10 times, 4 times a week. The percentage of load weight was gradually increased, 10% in the first 6 weeks and 12% in the last 6 weeks 18.

### Antibodies

Due to IHC and Wes-ProteinSimple, the following primary antibodies were used: primary rabbit polyclonal antibodies to Ki67 (GB111141, Servicebio), to CEACAM5 (DF6152, affinity), VEGFA (AF5131, affinity), VEGFC (DF7011, Affinity), Histone H3 (AF0863, affinity), ATP1A1 (AF6083, affinity), collagen type I (Proteintech, 14695-1-AP), MMP2 (GB11130, Servicebio), MMP9 (AF0220, Affinity), vimentin (AF7013, Affinity), TGF-β1 (AF7013, Affinity), E-cadherin (DF7157, Affinity), N-cadherin (AF5239, Affinity), rabbit monoclonal antibodies to TTF-1 (ab133737, abcam), to CD105 (ab221675, abcam) and mouse monoclonal antibodies to β-acting (sc47778, santa), to p63 (GB14129, Servicebio). Moreover, primary antibodies were used in combination with HRP-conjugated secondary antibodies (Jackson).

### IHC of paraffin sections

Mouse lung tumor tissues were made into paraffin-embedded sections. HE staining was performed using standard procedures. After staining, sections were placed under a microscope (Nikon Eclipse E100, Tokyo, Japan) for observation. For IHC analysis, paraffin sections were stained with antibodies according to the protocol provided by the manufacturer. Image analysis uses Image-Pro Plus 6.0 software.

### Wes automated western blotting system

Tissue protein extraction was performed using membrane, nuclear and cytoplasmic protein extraction kit (Sangon Biotech, China) according to the kit instructions. The separated cytoplasm, nucleus, and cell membrane proteins were used by the BCA protein assay kit to determine protein concentration (Beyotime, China). The samples were detected using the WesTM automatic protein expression analysis system (ProteinSimple, USA), and the specific steps were carried out following the instrument manual. The nuclear reference protein is Histone H3, the membrane reference protein is ATP1A1, and the cytoplasmic reference protein is β-acting.

### Statistical analysis

Independent Student's t-test was used for the data collected only from Saline group and CON group. Statistical analysis for multiple comparisons was performed in GraphPad Prism 7 using a one-way ANOVA followed by Dunnett's many-to-one test. The data were represented by mean ±SEM. Note: &, &&, &&&& respectively represent P<0.05, P<0.01, P<0.0001 compared with Saline group. *, **represent P<0.05 and P<0.01 compared with CON group.

## Results

### Moderate intensity constant load exercise (MICE) delays tumor progression

Lung cancer patients lose weight. For example, 66 patients with advanced bronchogenic cancer with different histological types lost more than 10% of their normal body weight 19. Indeed, we found that the weight of the mice in the Saline group was significantly higher than that in the CON group, MICE group, and HIIE group. Although the body weight of the mice in the MICE group was lower than that in the CON group from the 25^th^ to the 36^th^ week, the mice in the MICE group were slightly higher than the CON group at the 37th week. Moreover, the body weight of mice in the HIIE group was lower than that in the CON group from 27 weeks to 37 weeks (Figure S1A). In addition, the diet and water consumption of the mice in the Saline group were significantly higher than those in the other three groups. Compared with the CON group, the diet and water consumption of the mice in the MICE group improved (Figure S1B, C). These results also just confirm that the calorie intake of lung cancer patients is too low, which may be the cause of weight loss in lung cancer patients. It is worth noting that MICE can effectively improve the loss of appetite in lung cancer mice, and is beneficial for the maintenance of normal body weight.

**Figure 1:**
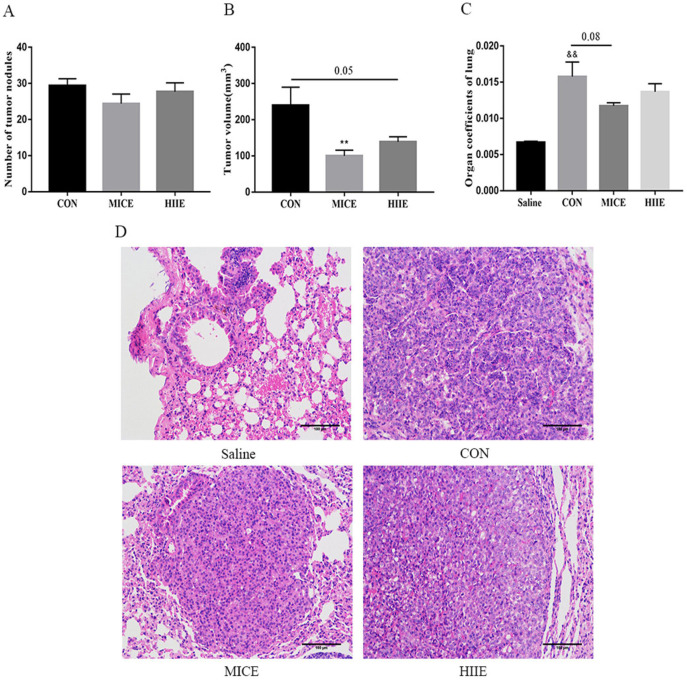
** The effect of exercise on tumor growth in mice with lung cancer. A, B.** The effect of exercise on the number of tumor nodules (A) and tumor size (B) in mice with lung cancer (n=10). **C.** The effect of exercise on lung tissue organ coefficient (n=8). **D.** The pathological changes of lung cancer tissues were observed using HE staining (scale bar, 100 µm). Note: && represents a significant difference compared with the Saline group (P<0.01).

In terms of the incidence of lung cancer, all 10 mice developed lung cancer at the 25th week after administration of urethane, while the Saline group that was given the saline injection at the 37th week did not develop lung cancer. In addition, all mice in the CON, MICE, and HIIE groups injected with urethane developed lung cancer. In terms of mortality, the CON group died during the entire modeling process, with a mortality rate of 16.7%. However, no mice died in the Saline group, MICE group, and HIIE group (Figure S2). Regarding the number of tumor nodules, there was no significant difference between the groups (Figure 1A). Notably, compared with CON, the size of lung tumors in the MICE group was significantly reduced (P<0.01). In addition, HIIE is inclined to reduce the size of lung tumors (P=0.05) (Figure 1B). Moreover, the organ coefficient of lung tissue in the MICE group decreased (P=0.08) (Figure 1C). From the results of HE staining, the bronchi and alveoli of the lung tissue of the Saline group are visible, while the bronchi and alveoli of the lung tissue of the CON group are no longer visible and replaced by a large number of inflammatory cells and tumor cells. In addition, compared with the CON group, the lung tissue tumor infiltration of the mice in the MICE group was alleviated, with some visible alveolar structures. However, although the tumor cell infiltration in the lung tissue of the HIIE group was relieved, it was not obvious (Figure 1D).

### Exercise does not change the level of tumor markers in lung cancer tissue

**Figure 2:**
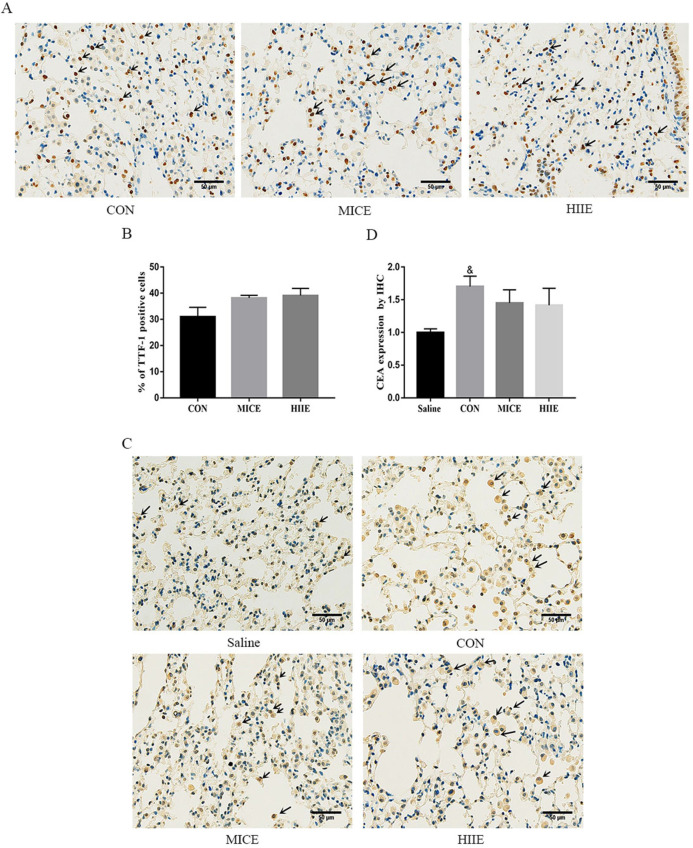
** The effect of exercise on the level of tumor markers in lung cancer tissue. A.** TTF-1 IHC staining. The arrow indicates the positive area (scale bar, 50 µm). **B.** Statistics of the percentage of TTF-1 positive cells (n=3). **C.** CEA IHC staining. The arrow indicates the positive area (scale bar, 50 µm). **D.** Statistical analysis of CEA expression (n=3). Note: & represents a significant difference compared with the Saline group (P<0.05).

Thyroid transcription factor-1 (TTF-1), a transcription factor, is normally expressed in thyroid epithelial cells and lung epithelial cells 20. It has been found that the crucial difference between lung adenocarcinoma and lung squamous cell carcinoma is the expression of TTF-1 and p63, in which lung adenocarcinoma is positive for TTF-1, while lung squamous cell carcinoma is positive for p63 21. Carcinoembryonic antigen (CEA) is among the markers of lung adenocarcinoma, and its expression is intimately related to the pathological progress and prognosis of lung cancer 22. The immunohistochemistry (IHC) results of this study showed that the lung cancer tissues of the CON group were positively stained for TTF-1, CEA was positively stained, and P63 was negatively stained (Figure S3A), which indicates that the lung cancer induced by urethane is lung adenocarcinoma. Since the elevated expression of TTF-1 is associated with better survival of lung cancer patients 23, our study counted the percentage of TTF-1 positive cells in each group and tested its protein expression level in the lung. Compared with the CON group, there was no difference in the percentage of TTF-1 positive cells between the MICE group and the HIIE group (Figure 2A, C). The results of Wes-ProteinSimple system showed that compared with the Saline group, the expression level of nuclear TTF-1 in lung tissue of lung cancer mice was significantly increased (P<0.05), but there was no significant difference in the level of nuclear TTF-1 between the CON group and the MICE group and the HIIE group (Figure S3B, C). In addition, since the expression level of CEA is also involved in the pathological stage of cancer, our study also detected the expression level of CEA in lung cancer tissues. Compared with the Saline group, the expression level of CEA in the CON group was significantly increased (P<0.05), but there was no significant difference in the expression of CEA between the CON group and MICE and HIIE (Figure 2B, D).

### Both MICE and high-intensity interval exercise (HIIE) reduce the percentage of Ki67-positive cells in lung cancer tissues

**Figure 3:**
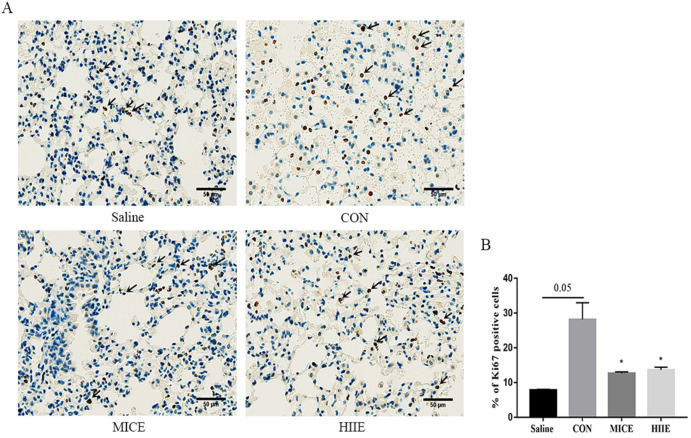
** The effect of exercise on the proliferation of lung cancer tissue cells. A.** Ki67 IHC staining. The arrow indicates the positive area (scale bar, 50 µm). **B.** Statistics of the percentage of ki67-positive cells in the lung tissues of mice in each group (n=3). Note: * represents a significant difference compared with the CON group (P<0.05).

To appreciate the mechanism of MICE on delaying tumor growth, our study further performed IHC staining on Ki67 of lung cancer tissue. Ki67 is a nuclear antigen associated with proliferating cells, and its percentage of positive cells reflects the speed of tissue cell proliferation. Our results showed that compared with the Saline group, the percentage of Ki67 positive cells in lung cancer tissues in the CON group had an increasing trend (P=0.05). Compared with the CON group, MICE and HIIE can significantly reduce the percentage of Ki67 positive cells in lung cancer tissues (P<0.05) (Figure 3A, B).

### MICE and HIIE delay tumor progression and do not depend on blood vessels and lymph vessels in tumor tissues

**Figure 4:**
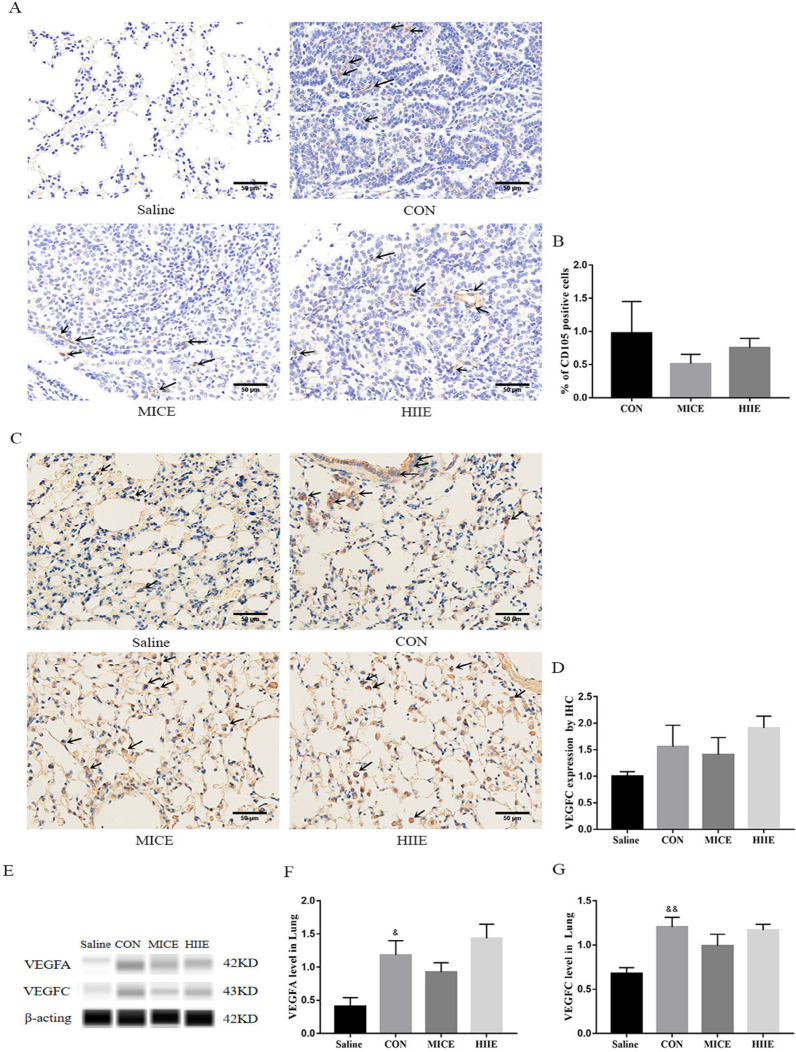
** The effect of exercise on lung cancer tissue angiogenesis and lymphangiogenesis. A.** CD105 IHC staining. The arrow indicates the positive area (scale bar, 50 µm). **B.** Statistics of the percentage of CD105-positive cells in the lung tissues of mice in each group (n=3). **C.** VEGFC IHC staining. The arrow indicates the positive area (scale bar, 50 µm). **D.** Statistics of VEGFC expression level in each group of IHC (n=3). **E, F.** The effect of exercise on the expression of VEGFA in lung cancer tissues (n=6). **E, G.** The effect of exercise on the expression of VEGFC in lung cancer tissues (n=6). Note: &, && respectively represent a significant difference compared with the Saline group (P<0.05), (P<0.01). *Represents a significant difference compared with the CON group (P<0.05).

Angiogenesis in tumor tissues can provide adequate nutrition for tumor cells. Moreover, neovascularization and lymphangiogenesis in tumors are also intimately involved in tumor metastasis. Thus, to assess whether the mechanism of exercise inhibiting tumor growth and metastasis is associated with tumor angiogenesis and lymphangiogenesis, we detected the protein expression of CD105, Vascular endothelial growth factor-A (VEGFA), and VEGFC lung cancer tissues. CD105 is a marker of vascular endothelial cell proliferation, which can effectively reflect the level of vascular proliferation in tissues. Indeed, the results of IHC showed that the healthy lung tissues of the Saline group showed negative expression of CD105, and the lung cancer tissues of the CON group showed positive expression of CD105. However, both MICE and HIIE had a tendency to the percentage of CD105-positive cells, but these changes are not statistically significant (Figure 4A, B). To assess the changes in the expression level of CD105 in lung cancer tissues, we used the Wes-ProteinSimple system for analysis. The results showed that there was no significant difference in CD105 expression between the groups (Figure S4A, B). However, it is worth noting that compared with the Saline group, the expression level of VEGFA protein in the CON group was significantly increased (P<0.05) (Figure 4E, F), which is beneficial to tumor growth and metastasis. In addition, the results of IHC showed that compared with the Saline group, the expression of VEGFC in the CON group had a rising trend, but it was not statistically significant (Figure 4C, D). It is worth noting that the results of the Wes-ProteinSimple system showed that, compared with the Saline group, the expression level of VEGFC in the CON group was significantly higher (P<0.01). However, neither the MICE group nor the HIIE group could reduce the expression level of VEGFC (Figure 4E, G), which means that MICE and HIIE cannot inhibit lymphangiogenesis in lung cancer tissues.

### HIIE may promote tumor EMT through TGF-β1

**Figure 5:**
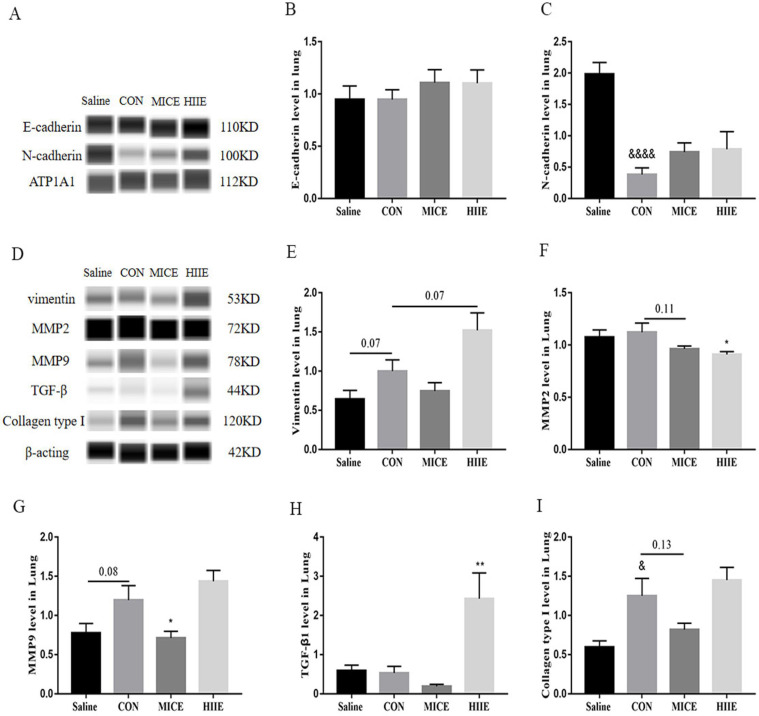
** The effect of exercise on EMT and its inducing factors in lung cancer tissues. A, B.** The expression level of E-cadherin in lung cancer tissues (n=6). **A, C.** N-cadherin expression level in lung cancer tissues (n=6). **D, E.** The expression level of vimentin in lung cancer tissues (n=6). **D, F, G.** The expression levels of MMP2 (D, F) and MMP9 (D, G) in lung cancer tissues (n=6). **D, H.** The expression level of TGF-β1 in lung cancer tissues (n=6). **D, I.** The expression level of collagen type I in lung cancer tissues (n=6). Note: &, &&&& respectively represent a significant difference compared with the Saline group (P<0.05), (P<0.0001). *, ** respectively represent significant differences compared with CON group (P < 0.05), (P < 0.01).

Because the process of tumor EMT is closely related to tumor cell metastasis and invasion, we detected E-cadherin and N-cadherin in tumor tissues. We found that there was no significant difference in the expression of E-cadherin between the groups (Figure 5A, B). However, surprisingly, compared with the Saline group, the expression of N-cadherin in the CON group was significantly reduced (P<0.0001) (Figure 5A, C). In most tumors, the expression of N-cadherin is elevated during EMT, which helps to enhance the motility and aggressiveness of tumor cells. Therefore, we further tested the expression level of vimentin, a mesenchyme marker. The results showed that compared with the Saline group, the expression level of vimentin in the CON group had a tendency to increase (P=0.07) (Figure 5D, E), which means that the occurrence of EMT may not necessarily be accompanied by the up-regulation of N-cadherin, and the reason may be due to the heterogeneity of the tumor. In addition, compared with the CON group, the expression level of vimentin in the HIIE group also had a tendency to increase (P=0.07), which indicates that HIIE tends to induce EMT in tumor cells. Given that MMP2, MMP9, TGF-β1, and collagen type I were mentioned above to be associated with EMT in tumor cells, the expression of these molecules was further examined to further explore the mechanism of HIIE-induced EMT in tumors. Compared with the Saline group, the expression of MMP9 in the CON group tended to increase (P=0.08). Importantly, compared with the CON group, the expression level of MMP9 in the MICE group was significantly lower (P<0.05) (Figure 5D, G). In addition, compared with the CON group, the expression of MMP2 in the HIIE group was significantly reduced (P<0.05) (Figure 5D, F), which means that MICE and HIIE can inhibit the metastasis of tumors by regulating the expression of MMP9 and MMP2, respectively. Moreover, compared with the CON group, the level of TGF-β1 in the HIIE group was significantly increased (P<0.01) (Figure 5D, H), which means that HIIE may induce EMT by activating TGF-β1. In addition, the expression of collagen type I in the CON group was significantly increased (P<0.05). However, neither MICE nor HIIE can reduce the expression of collagen type I (Figure 5D, I).

## Discussion

Numerous studies revealed that exercise can delay the progression of tumors. For example, aerobic exercise can effectively suppress the tumor progression in lung cancer mouse model constructed by A549 xenografts 24. After the intervention of aerobic exercise, lung cancer patients' symptoms have also been relieved 4. Moreover, voluntary running also suppresses tumor growth 25. These studies have confirmed that aerobic exercise has a certain therapeutic effect on tumors. It was also found that high-intensity interval exercise, but not aerobic exercise, diminishes the incidence of lung cancer in mice more 5, probably by inducing lactate to inhibit glycolysis in tumor tissues 26. Furthermore, high-intensity interval exercise mitigates tumor growth by increasing the number of circulating NK cells and the activity of NK cells 27. These studies seem to indicate that high-intensity interval exercise also has good anti-tumor effects. Indeed, we found that both MICE and HIIE reduced the mortality of lung cancer mice and prolonged the survival time of lung cancer mice (Figure S2). In addition, although MICE and HIIE did not alter the number of tumor nodules in the lungs of mice, MICE can significantly reduce the tumor volume and HIIE also has a tendency to reduce tumor volume (Figure 1A, B). Moreover, it is worth noting that MICE had a tendency to reduce the increased organ coefficient of lung tissue in mice with lung cancer, but the HIIE had no such effect (Figure 1D). Furthermore, the results of HE staining showed that the size of alveoli and bronchi in the lung tissue of healthy mice were clearly visible, but the alveoli and bronchi of lung cancer mice were no longer visible, and there were a large number of tumor cells and inflammatory cell infiltration. However, after MICE and HIIE, the tumor mass of lung cancer tissue of mice decreased to varying degrees, but the decrease in MICE was even more obvious. It is worth noting that the amount of exercise in HIIE in this study is quite small compared to MICE. Therefore, it is also possible that HIIE is less effective than MICE in the treatment of lung cancer due to the small amount of exercise. It is important to note that the exercise intensity of HIIE is high, and patients with cancer or other diseases may not be able to perform prolonged HIIE. In addition, some studies have found that low-volume HIIE can also have a good exercise effect. In fact, low-volume HIIE significantly enhance adolescent cardiorespiratory fitness and significantly reduce adiposity 28. In addition, low-volume HIIE has stronger effect on energy expenditure and excess post-exercise oxygen consumption in the early post-exercise recovery period compared to MICE 29. These studies imply that the volume of exercise may not be the key to the health effects of HIIE. Thus, based on the above results, it can be concluded that MICE is better for the treatment of lung cancer than HIIE. Besides, the combination of multiple exercise methods can also have a positive therapeutic effect on cancer patients. For example, it has been found that a three-month modular multi-modal physical exercise program, including high-intensity resistance training, moderate intensity endurance training, and low-intensity stretching activities, delay or prevent skeletal complications and improve physical function in prostate cancer patients with bone metastases 30. This suggests that the combination of a variety of exercises of different intensities and different exercise methods may have equally positive effects on the treatment of lung cancer, which merits further exploration in the future.

It has been observed that the expression of TTF-1 is associated with better survival in patients with advanced lung adenocarcinoma 23. Mechanistically, TTF-1 inhibits the EMT in lung adenocarcinoma cells mediated by TGF-β1 31. Moreover, the expression of TTF-1 in lung adenocarcinoma tissue is higher than that in normal tissues adjacent to cancer 32. Indeed, we found that the expression level of TTF-1 in lung cancer tissue is higher than that in normal lung tissue (Figure 2A, B) (Figure S3B, C). Unfortunately, neither MICE nor HIIE was able to change the level of TTF-1 in lung cancer tissues. In addition, studies have found that elevated CEA expression levels in tumor tissues indicate a poor prognosis 33, and the high level of CEA in the serum of lung cancer patients is closely connected with the shorter survival period 34. Similarly, IHC results showed that MICE and HIIE also failed to reduce the elevated CEA levels in lung cancer tissues (Figure 2C, D).

Tumor growth is closely connected with Ki67 and angiogenesis-related factors. However, endurance exercise can reduce the expression of Ki67 and angiogenesis factors VEGF and CD31 in tumor tissues 35, which suggests that MICE may affect the proliferation of tumor cells by reducing angiogenesis in tumor tissues. It is worth noting that CD105 is overexpressed in actively proliferating vascular endothelial cells, and it has been confirmed to be highly expressed in blood vessels in tumor tissues. Intratumoral microvessel density (MVD) determined using CD105 antibody has been found to be an independent prognostic indicator, wherein increased MVD correlates with shorter survival 36. In addition, the high expression of Ki67 in tumor tissues also indicates a high proliferation level of tumor cells and a poor prognosis. The results of IHC showed that both MICE and HIIE reduced the percentage of Ki67-positive cells in lung cancer tissues, indicating that both exercises can inhibit the proliferation of lung cancer cells. To explore whether its mechanism is relevant to angiogenesis, this study further examined the changes in the expression of relevant vascular markers. Indeed, we found that healthy lung tissue was negative for CD105, while lung cancer tissue was positive for CD105. Unfortunately, neither MICE nor HIIE can depress the percentage of CD105-positive cells in lung cancer tissues (Figure 4A, B). Moreover, the VEGFA can induce migration and proliferation of vascular endothelial cells, and then promote angiogenesis in tissues 37. We found that VEGFA in lung tissue of lung cancer mice is significantly higher than that in healthy mice, which means that it is beneficial to angiogenesis and growth of lung cancer cells in lung cancer tissues. It has been observed that there is a positive correlation between the expression of VEGFA and CD105 38. Indeed, the results of this study are consistent with their results, which indicates that increased angiogenesis in lung cancer tissues is beneficial to the growth of tumor cells. Moreover, angiogenesis can not only provide abundant nutrition for tumor tissues, but also facilitate the metastasis of tumor cells. In addition to metastasis through blood vessels, tumor cells can also metastasize through lymphatic vessels. It has been found that the expression of VEGFC in tumor tissues is beneficial to tumor metastasis 39. Indeed, we found that the expression of VEGFC in lung cancer tissues was significantly higher than that in healthy lung tissues. In addition, it has been found that long-term endurance exercise delays the growth of breast cancer by inhibiting the expression of VEGFA in tumor tissues 40. Unfortunately, we found that neither MICE nor HIIE reduced the expression of VEGFA and VEGFC in lung cancer tissues (Figure 4E, F, G), indicating that neither MICE nor HIIE control proliferation and metastasis of tumor cells by angiogenesis and lymphangiogenesis in tumor tissues.

The EMT process, regulated by many factors such as MMPs, TGF-β1, and collagen type I, triggers the migration and invasion of tumor cells 8-11, 41. For example, VEGF enhances the expression of EMT markers N-cadherin, vimentin, and MMP2 and MMP9, reduces the expression of E-cadherin, promoting the invasion and migration of nasopharyngeal carcinoma cells 42. Mounting evidence has shown the expression of E-cadherin in most tumor cells is decreased, while the expression of N-cadherin is increased. For example, studies have found low expression of E-cadherin, high expression of N-cadherin, high expression of vimentin and MMP-9 in lung adenocarcinoma 43. However, we found that compared with healthy lung tissue, the expression of E-cadherin in lung cancer tissue did not decrease, and the expression level of N-cadherin was significantly reduced (Figure 5A, B, C). In fact, it has been found that some breast cancer cell lines express high levels of E-cadherin and low levels of N-cadherin, while some breast cancer cell lines express the opposite 44. In addition, decreased expression of N-cadherin is found in human osteosarcoma, ovarian cancer, malignant glioma, and renal cell carcinoma, which is connected with the further spread of tumors 45. Moreover, it is found that 13 of 14 lung adenocarcinoma patients were positive for E-cadherin and negative for N-cadherin 46. Furthermore, among 29 lung cancer samples, 18 cases of membranous and cytoplasmic N-cadherin expression increased, and 11 cases did not change 47. Finally, among 47 NSCLC patients, some patients preserve the expression of E-cadherin 48. Therefore, these studies mean that the expression of E-cadherin and N-cadherin in lung cancer tissues is extremely complex, and these inconsistent changes may be derived from tumor heterogeneity. Based on the above analysis, the down-regulation of N-cadherin in lung cancer tissues in our study may be tied to the enhancement of its invasion ability. Unfortunately, neither MICE nor HIIE altered E-cadherin and N-cadherin in lung cancer tissues. However, it is worth noting that the expression of vimentin, the EMT marker, in lung cancer tissues tends to increase (P=0.07), while HIIE tends to increase the expression of vimentin (P=0.07) (Figure 5D, E), which indicates that HIIE tends to enhance EMT in lung cancer cells. In addition, we found that HIIE significantly increased the expression of TGF-β1 in lung cancer tissues, which also means that HIIE may stimulate the EMT in lung cancer cells through TGF-β1. Moreover, TGF-β1-activated Smad3/4 complex transcriptionally upregulates N-cadherin expression in non-small cell lung cancer 49. However, this is inconsistent with the result that HIIE did not change N-cadherin, and the exact mechanism still needs to be elucidated. It has been observed that the invasion and metastasis of lung adenocarcinoma are intimately related to MMP2 and MMP-9 50, which can degrade ECM for tumor invasion and initiation of EMT 51. We found that although the expression of MMP2 in lung cancer tissues did not increase, the expression level of MMP9 tended to increase (P=0.08). Strikingly, MICE and HIIE markedly reduced the expression of MMP9 and MMP2 in lung cancer tissues, respectively (Figure 5D, F, G), which means that MICE may restrain the metastasis of lung cancer cells by decreasing the level of MMP9. In addition, regardless of the fact that HIIE reduces the level of MMP2 in lung cancer tissues, it may promote the EMT of lung cancer cells. Unfortunately, our results show that exercise does not reduce the elevated collagen type I in lung cancer tissues, which also means that neither MICE nor HIIE can regulate the EMT of lung cancer cells through collagen type I (Figure 5D, I). In general, our work, for the first time, compared the effect of MICE and HIIE on tumor growth in mice with lung cancer and discovered the mechanism by which HIIE is not as effective as MICE in treating lung cancer.

## Supplementary Material

Supplementary figures.

## Abbreviations

EMT: epithelial-mesenchymal transition
MMP2/9: matrix metalloproteinases 2/9
TGF-β1: transforming growth factor-β1
MICE: moderate intensity constant load exercise
HIIE: high-intensity interval exercise
ECM: extracellular matrix
TTF-1: thyroid transcription factor-1
CEA: carcinoembryonic antigen
VEGFA: vascular endothelial growth factor-A
VEGFC: vascular endothelial growth factor-C
ATP1A1: Na+/K+ ATPase α1
