# Supplementary material for: Compared with High-intensity Interval Exercise, Moderate Intensity Constant Load Exercise is more effective in curbing the Growth and Metastasis of Lung Cancer
Source: J Cancer. 2022 Feb 28;13(5):1468–79. doi: 10.7150/jca.66245 (PMC8965116; doi:10.7150/jca.66245)
Supplement: Supplementary file 1 — Supplementary figures. [file jcav13p1468s1.pdf]

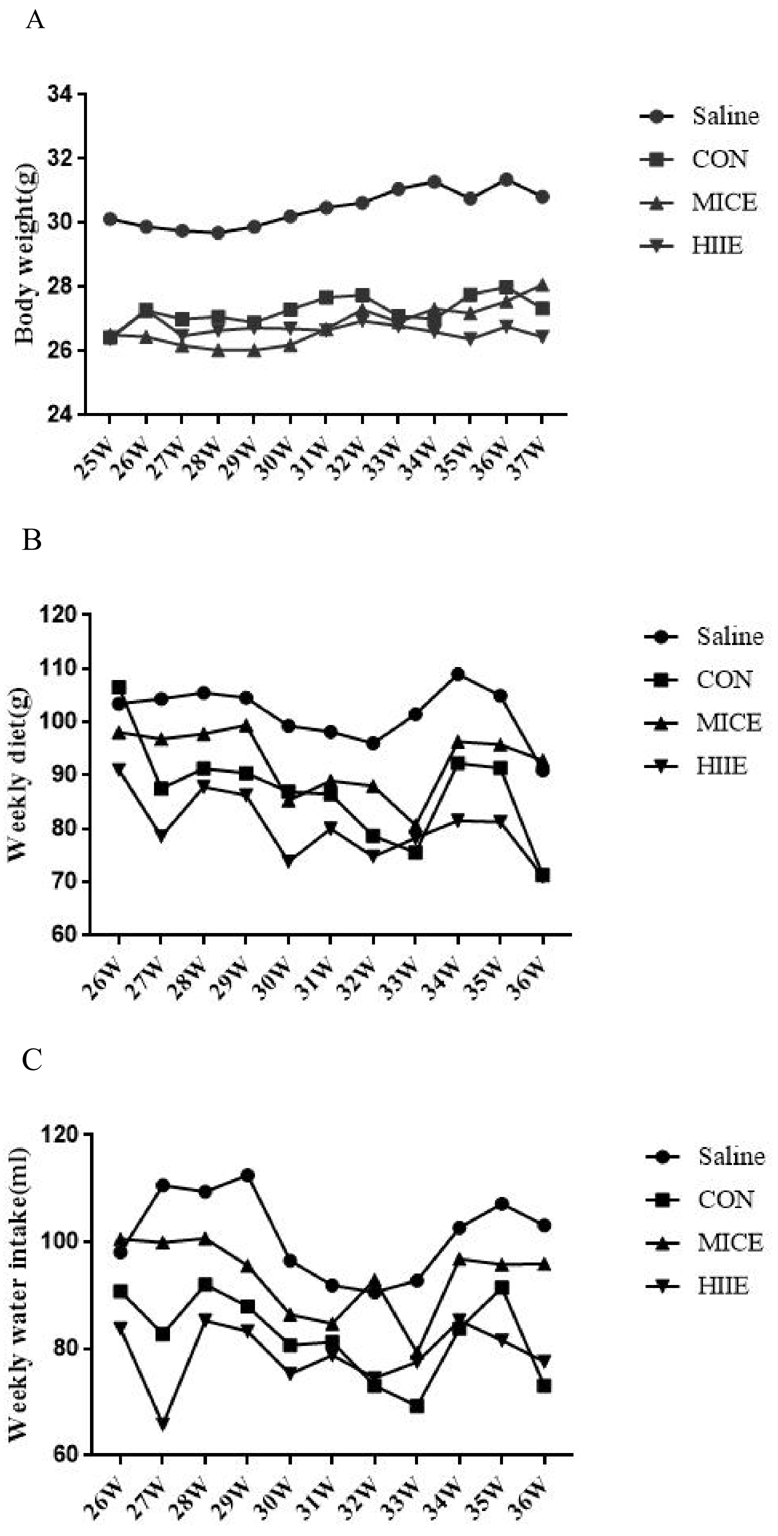

Figure S1: Changes in body weight and drinking water volume of mice in each group. A: Weekly weight changes of mice in each group (n=8). B: Changes in the weekly diet of mice in each group. C: Changes in the weekly drinking water of mice in each group.

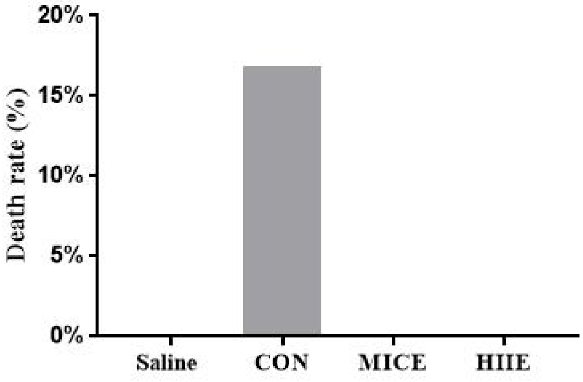

Figure S2: Mortality of mice in each group

A

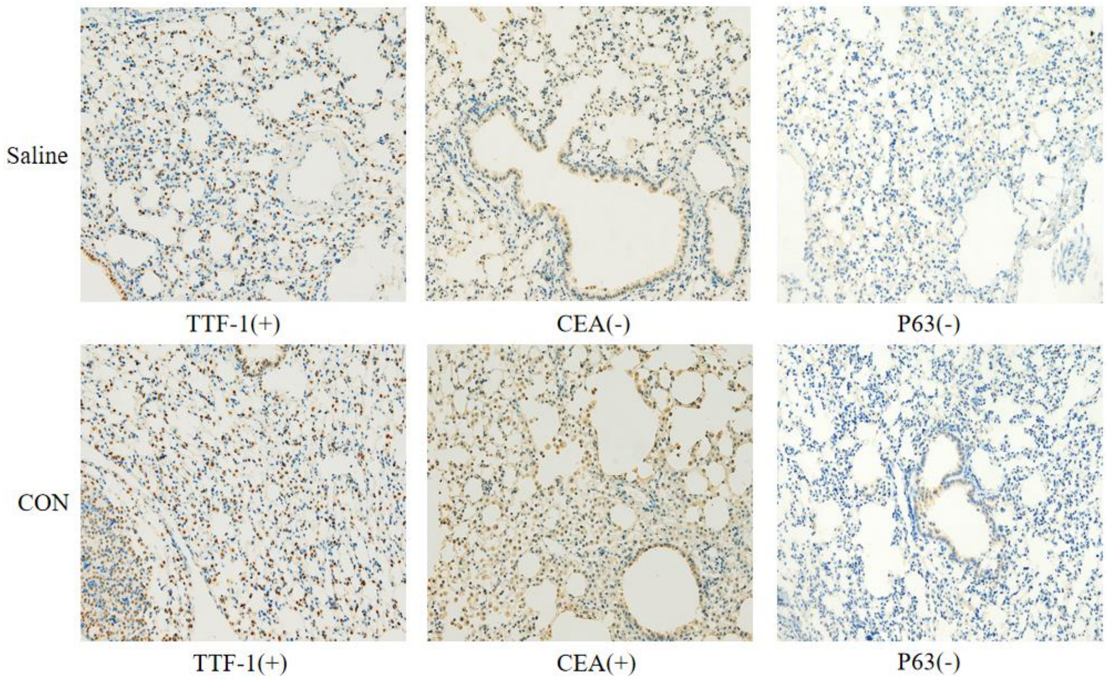

B C

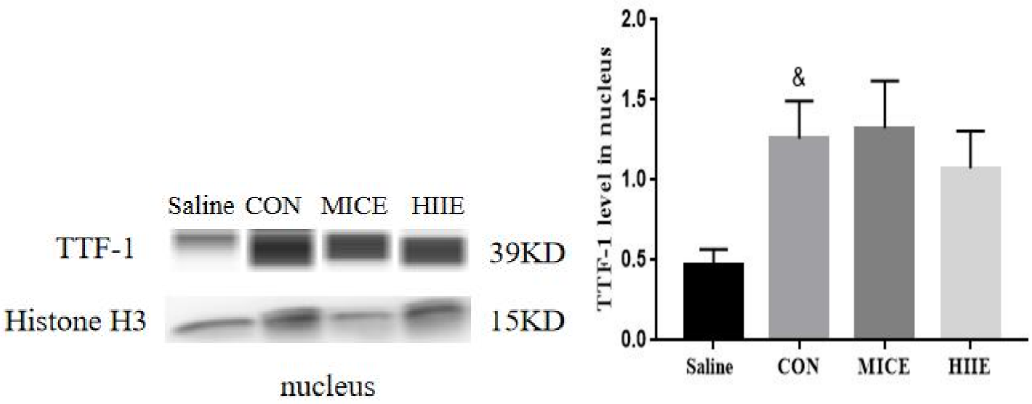

Figure S3: A: IHC staining of lung cancer tissues TTF-1, CEA and P63 (n=3). B, C: Effect of nuclear TTF-1 expression in lung tissue of mice with lung cancer (n=6).

A B

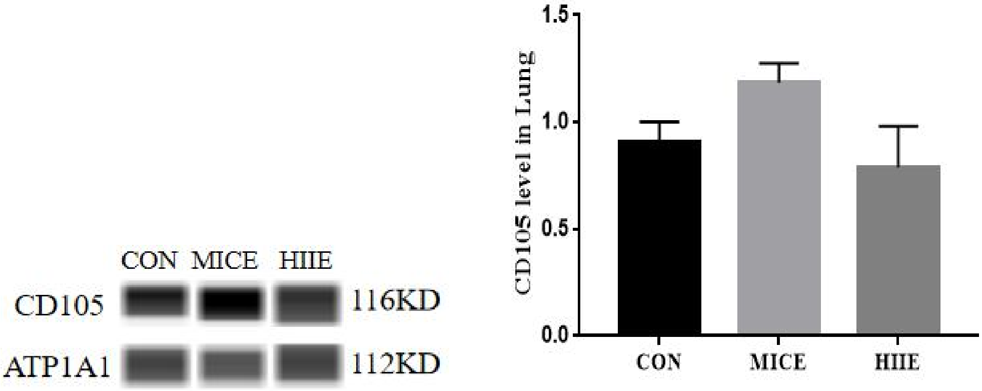

Figure S4: The expression level of CD105 in lung cancer tissues (n=6).
